# Assessment of Eutrophication and DOC Sources Tracing in the Sea Area around Dajin Island Using CASI and MODIS Images Coupled with CDOM Optical Properties

**DOI:** 10.3390/s21144765

**Published:** 2021-07-13

**Authors:** Shuchang Ma, Xiaoyu Zhang, Yixuan Xiong, Guorong Huang, Yachao Han, Valerio Funari

**Affiliations:** 1 School of Earth Sciences, Zhejiang University, Hangzhou 310027, China; shuchangma23@163.com (S.M.); bravedoris1994@163.com (Y.X.); 18787189707@163.com (G.H.); 2 Ocean Academy, Zhejiang University, Zhoushan 316000, China; 3 China Aero Geophysical Survey and Remote Sensing Center for Natural Resources, Beijing 100083, China; hanyachao@mail.cgs.gov.cn; 4 Institute of Marine Science (ISMAR), National Research Council (CNR), via P. Gobetti 101, 40129 Bologna, Italy; valerio.funari@bo.ismar.cnr.it

**Keywords:** airborne and aerospace remote sensing, aquatic eutrophication assessment, dissolved organic carbon (DOC), chromophoric dissolved organic matter (CDOM), satellite chlorophyll

## Abstract

The sea area around Dajin Island in the Pearl River Estuary is the second-largest habitat in China for the Indo-Pacific humpback dolphin (Sousa Chinensis). However, the rapid economic development of this area brings potential threats to the aquatic ecology around Dajin Island. Real-time monitoring and evaluation of the ecological health of the sea area are urgent. In this study, band ratio and single-band inversion algorithms were performed to obtain Chlorophyll-a (Chl-a) and Suspended Sediment Concentration (SSC), relying on both Compact Airborne Spectrographic Imager (CASI) and Moderate resolution Imaging Spectrometer (MODIS) images. The CASI/Chl-a with high spatial resolution was adopted to assess the eutrophication status, while the dissolved organic carbon (DOC) concentration and chromophoric dissolved organic matter (CDOM) optical properties were used to derive the material composition and sources. The results suggest that the study area is under a low to medium eutrophication state with evenly distributed low Chl-a concentration. However, higher Chl-a is observed in the outer estuary with MODIS/Chl-a. The relatively high DOC concentration, especially in the north, where aquaculture is practiced, and near the estuary’s main axis, i.e., east Dajin Island, indicates that the eutrophication state might be underestimated using satellite chlorophyll alone. CDOM optical properties indicated that terrestrial materials are the DOC’s primary material sources, but the DOC derived from fishery aquaculture cannot be ignored. The low Chl-a concentration is likely due to the turbulent hydrodynamic regime caused by jet flow and reciprocating flow in this marine area. Comprehensive observation, including the assessment of different technological platforms, is suggested for the aquatic environment.

## 1. Introduction

Dajin Island is located in the Huangmaohai Sea, Pearl River Estuary, China. The Huangmaohai Sea is the estuary of the Xijiang River, one of the main second-order estuaries located at the southernmost of the Pearl River Estuary. The sea area around Dajin Island is the second-largest habitat in China for the Indo-Pacific humpback dolphin (Sousa Chinensis), a national first-class protected marine mammal in China [1]. The natural reserve area reaches 107.477 km^2^, with about 280 Indo-Pacific humpback dolphins with a multigenerational structure [2], and it is also a key area for “habitat backup” of the Indo-Pacific humpback dolphin population [3]. However, with the rapid expansion of industrialization and urbanization in the Pearl River Delta, the water quality of the Huangmaohai Sea is worse than category Ⅳ according to the seawater quality standard, China (GB 3097-1997) (Environment Quality Report of Guangdong Province, China, 2017, 2018, 2019). The main pollutants are inorganic nitrogen and reactive phosphorus. The semi-enclosed topography and tidal flow of the Pearl River Estuary aggravated the accumulation of nutrients in the seawater, incurring frequent red tides [4], which threatens the survival of the Indo-Pacific humpback dolphin [5,6,7]. Therefore, in order to alert the potential ecological risks of the Indo-Pacific humpback dolphin, it is very important to develop a way to monitor and comprehensively evaluate the environmental quality and ecological health in a timely manner, and to identify the sources of pollutants and influencing factors. However, at present, only conventional routine water quality monitoring around Dajin Island is performed based on observation from sparse stations. Few studies have examined the habitat environment in details in this sea area, in comparison with other dolphin habitats, including the Hongkong Sea [8] in the northern Pearl Estuary and Beibu Bay [9] in the south of Hainan Island in the South China Sea. The lack of knowledge of the formation and ecological effects of eutrophication may mislead the policy-making for local aquatic environment protection. Recently, observation technology has largely developed for vast ocean observation, with satellite remote sensing solutions being one of the most prominent achievements [10]. Satellite ocean color remote sensing can obtain information such as sea surface temperature [11], Chl-a [12,13], SSC (Suspended Sediment Concentration) [14] and CDOM (Chromophoric Dissolved Organic Matter) [15,16]. Studies have shown the capability of CDOM in quantitatively estimating DOC in river estuaries, coastal waters and upwelling areas [17]. This is especially significant in the rapid assessment of DOC flux in the marginal sea [18,19], and the value of CDOM as a tracer of man-made organic pollutants has also been revealed [20]. Up to date, Moderate resolution Imaging Spectrometer (MODIS), MERIS (Medium Resolution Imaging Spectrometer Instrument), HY-1 and GOCI (Geostationary Ocean Color Imager) are widely used to achieve long time series images with wide coverage and high frequency, which is of great significance for currents observation, primary productivity estimation, eutrophication assessment, land-sea interaction, carbon flux estimation, etc. [21,22]. Moreover, aerial remote sensing technology creates more flexibility in emergency monitoring [22]. At present, the airborne imaging spectroscopy systems have been widely used in coastal monitoring, especially for oil spill, red tide and other purposes [23]. However, using the information provided at different scales of observation as well as gathering different parameters from different platform observation technologies into a harmonized and operable data-sharing system is challenging in terms of workforce deployed and technical commitment [24,25] but vital to perform ocean observation with big data.

This study tried to assess the eutrophication status of the sea area around Dajin Island by combining CASI and MODIS images with measurements of DOC and CDOM optical properties. Chl-a concentration was determined based on CASI and MODIS, through which the regional eutrophication state and potential ecological risk were assessed. The inverted suspended sediment concentration (SSC) from both the CASI and MODIS are used to help observe the hydrodynamical environment and current movement. SSC is also an important factor determining the transparency and impacts the utilization of solar energy by algae. Moreover, DOC concentration, CDOM absorption properties, including (441) (absorption coefficient of CDOM at 441 nm) and Sg (slope parameter of CDOM absorption spectrum), CDOM fluorescence properties, including the fluorescence intensity at 441 nm (Fs(441)), and Excitation Emission Matrix spectroscopy (EEM) characteristics of CDOM decoupled with PARAFAC (parallel factor analysis) were used to trace the DOC sources.

This study aims to answer the following scientific questions:

(1) What is the eutrophic state in the sea area around Dajin Island?
(2) Is there any ecological risk of eutrophication in the study sea area?
(3) What are the main material sources related to Dissolved Organic Matter (DOM) in this area?

In addition, factors that may affect the relationship between DOC and CDOM are discussed. Finally, this work would be a critical verification to assess the feasibility of quantifying DOC by CDOM in a specific sea area and overall environmental status by remote sensing techniques coupled with relatively few direct measurements.

## 2. Hydrodynamic Characteristics

**Figure 1:**
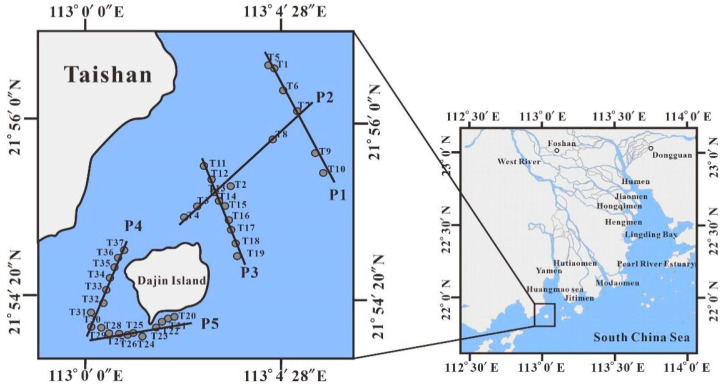
The geographical location of Dajin Island and its surrounding waters [27].

The studied sea area is approximately 39.318 km^2^ around Dajin Island (Figure 1) at 112°59′30″ to 113°04′00″ E and 21°46′00″ to 21°53′00″ N. It is located at the mouth of the trumpet-shaped morphology of the Huangmao Sea in the Pearl River Estuary. The study area is under control of a warm subtropical monsoon, with abundant rainfall; the annual total cloud cover can reach 60–70% [26].

The Pearl River Estuary is formed by eight rivers (Wujiang River, Niulanjiang River, Chishui River, Yuanjiang River, Nanpanjiang River, Beipanjiang River, Hongshui River, and Duliujiang River) transporting terrigenous materials from different basins to the estuary through eight tributaries or gates (Humen Gate, Jiaomen Gate, Hongqimen Gate, Hengmen Gate, Modaomen Gate, Jitimen Gate, Hutiaomen Gate, and Yamen Gate). Dajin Island is located at the mouth of the Yamen-Hutiaomen gate in the west part of the Pearl River Estuary (Figure 1). The Yamen Gate and Hutiaomen Gate are two main channels transporting terrestrial materials to the marine area around Dajin Island. It is worth noting that only the Yamen Gate and Hutiaomen Gate are tidal estuaries of the eight gates. The width of these two gates is nearly 10 times narrower than the width of the riverbed upstream and downstream, which induces a powerful jet effect under huge tidal capacity. The jet system causes very high flow velocity at the Yamen Gate and Hutiaomen Gate regardless of the rising or falling tide. The two-gate estuary system dramatically changes the hydrodynamic flow field formed by the tidal current alone [28]. The complicated hydrodynamic conditions and heterogenous terrigenous sources provide unique optical characteristics to the turbid water of the Pearl River Estuary [29].

## 3. Methods and Data

The data used in this study include the measured water surface spectra, i.e., CASI and MODIS images. Chl-a, SSC, DOC, salinity, water absorption, and fluorescence spectra were also measured on water samples collected at each site.

### 3.1. Remote Sensing Images

#### 3.1.1. Airborne CASI Image

Aerial hyperspectral remote sensing has been widely used in river, lake [30], and coastal observations [31]. In this study, the airborne image was acquired in a test flight conducted by the Aeronautical Geophysical Remote Sensing Center of the Ministry of Natural Resources of China from 15:00 to 17:00 on 11 July 2018, with a CASI-1500 VNIRh airborne imaging sensor produced by Itres (Canada). The CASI is designed with up to 288 channels, friendly customizable settings between bands ranging from 380 to 1050 nm, and capable of high spectral resolution that makes it suitable for advanced water monitoring. The spatial resolution of 0.6 m provided detailed information on localized sea areas. The technical parameters of CASI compared to those of MODIS are shown in Table 1.

**Figure 2:**
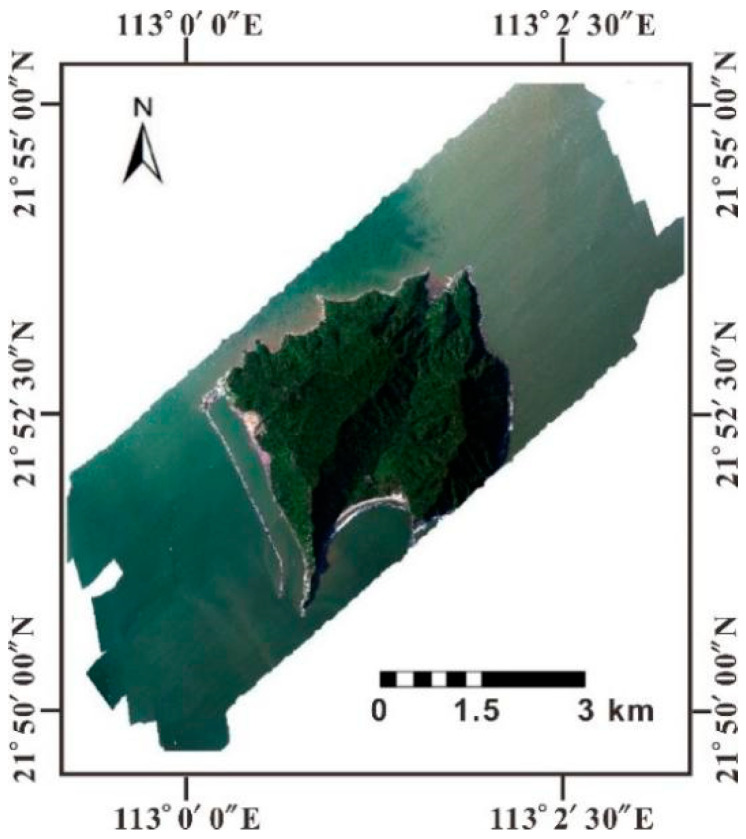
True color synthesis of the CASI data from the study area.

The flight altitude was 400 m. The acquired image was 5.27 km wide and 13.64 km long. The true color composite image (R636.791, G536.666, B436.583) is shown in Figure 2.

Preprocessing of the CASI image mainly included radiometric correction, geometric correction, image mosaic, atmospheric correction, and water body extraction. The geometric correction was based on the Global Positioning System (GPS)/Inertial Measurement Unit (IMU)-assisted aerial linear array push broom imaging technology. The system geometric error between the direct measurement value of airborne and the actual position, and attitude value of hyperspectral camera exposure was eliminated through the layout of the hyperspectral land calibration field [32]. The atmospheric correction was performed with the FLAASH atmospheric correction module of ENVI software, and the water body was extracted by the normalized difference water index (NDWI), threshold segmentation, and artificial discrimination [33].

#### 3.1.2. Aerospace MODIS Image

Although the CASI can provide subtle information with high spatial resolution, for the narrow spatial coverage, the MODIS image was selected as it provides more background information. In addition, the inversion algorithm established for the CASI can be transplanted to the MODIS.

**Figure 3:**
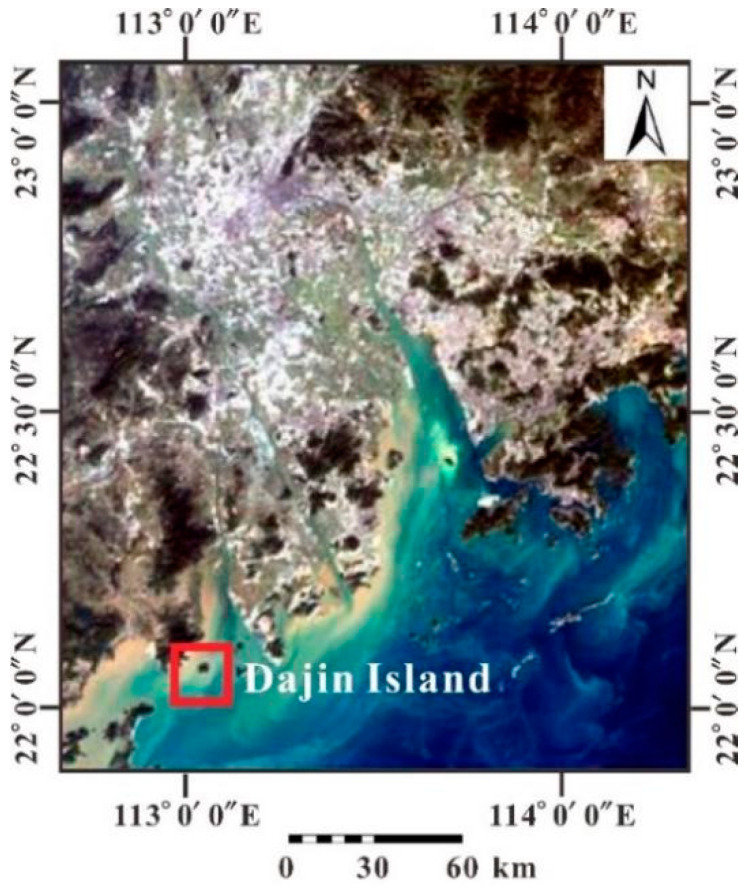
The tailored MODIS image which covers the Pearl River Estuary.

However, due to the cloudy weather and haze days in the study area, it is difficult to obtain good quality images, even though MODIS flies over twice a day. We failed to get MODIS images of good quality on the same day as the CASI. In order to minimize the impacts by satellite imaging taken at different times, we searched the archive to obtain MODIS images with good quality. The screening rules were as follows: (1) the MODIS images should be taken as close as possible to the time of the CASI; (2) the images should be taken on cloudless days over the study area to ensure the best quality; and (3) the MODIS and CASI were taken in the similar hydrodynamical environment. The MODIS image ((MOD02HKM.A20182060250.061.2018206133950) was finally selected; both the MODIS and CASI images were taken during the slack water after the hide tide. Geometric correction, radiometric calibration, and atmospheric correction were carried out in ENVI, and NDWI was used to extract the water body from the image [33]. As shown in Figure 3, the image above the study area is cloudless.

### 3.2. Station Locations and Sample Collection

Water sampling (during 24–25 October 2018) and sea surface optical measurements were performed during the same tide as the flight to reduce the changes in the distribution characteristics of water color parameters caused by changes in the hydrodynamic conditions of the estuary, that is, to reduce the differences due to non-synchronous measurements.

A total of 37 sampling stations were established in the sea area surrounding the island while considering the geographical location and corresponding hydrodynamic characteristics (Figure 1). Among them, 24 stations fell within the coverage area of the CASI images: 16 stations (T13, T15, T16, T18, T22, T20, T25, T26, T28, T29, T32, T33, T34, T35, T36, and T37) were selected among the 24 stations for the remote sensing inversion algorithm construction, while the remaining eight stations (T14, T17, T21, T23, T24, T27, T30, and T31) were used for accuracy verification. A total of 13 stations (T1, T2, T3, T4, T5, T6, T7, T8, T9, T10, T11, T12, and T19) were established to the east of Dajin Island near the main axis of the estuary for model construction of the MODIS image and accuracy verification. No station was established in the aquaculture area because the area is inaccessible.

The sampling campaign was conducted along five transects (P1–P5 in Figure 1): P1 is located east of the island, close to the Pearl River Estuary’s main axis; P2 is a NE-SW transect connecting P1 to the aquaculture area northeast of Dajin Island; P3 is parallel to P1, but closer to the island’s coast; P4 is located in the maritime area between the continental land and the island’s west coast; P5 is located south of Dajin Island where the main tourist harbor is located.

### 3.3. Measurements of DOC and CDOM Optical Properties

DOC represents the total dissolved organic matter in the water. In the coastal zone of the estuary, the DOC in the water is not only closely related to the photosynthesis of phytoplankton, biological metabolism, and bacterial activity, but is also an important parameter to characterize the content of organic matter and biological activity level in water. It is also closely related to terrestrial input humus and anthropogenic organic pollutants. Therefore, it is an important part to study the carbon cycle flux in the process of land sea interaction [18,19,20]. The DOC concentration was measured using the Shimadzu (Japan) TOC-L total organic carbon analyzer [34]. The high temperature catalytic oxidation method (HTCO) was used to oxidize the water sample to carbon dioxide with oxygen as the carrier gas, and the amount of carbon dioxide produced was quantified.

CDOM represents the colored part of the DOC [35]. The optical properties of CDOM have been proven to be useful in analyzing the material composition and sources of the DOC [35]. In this study, the CDOM optical properties were used to determine the DOC material sources. The CDOM absorption spectrum was measured using a Horiba Aqualog ultraviolet fluorescence spectrometer following the marine optical measurement specifications recommended by NASA. A 1 cm four-way quartz cuvette was used with Milli-Q water as a reference blank. Each sample was scanned three times within the wavelength range of 240–600 nm with a spectral resolution of 3 nm.

Typically, the absorption coefficient (α) at 355 nm, 400 nm, and 440 nm is used to indicate the concentration of CDOM, and the two are directly proportional [36]. In this study, α (441) was selected to determine the CDOM concentration because of its robust correlation (R^2^ = 0.68–0.88).

The spectral slope coefficient (Sg) is a qualitative index of CDOM, which can be related to the average molecular weight of the dissolved organic matter detected by CDOM measurements. The Sg value can be derived from the following formula:

(1) $$\alpha \left(\lambda \right)=\alpha \left(\lambda_{0}\right)\exp\left(S_{g}\left(\lambda_{0}-\lambda \right)\right)+K$$

where α(*λ*) represents the absorption coefficient at wavelength *λ*; α(*λ*_0_) represents the absorption coefficient at the reference wavelength *λ*_0_ (441 nm); and *K* is a background parameter used to reduce errors caused by instrument noise.

Fluorescent Dissolved Organic Matter (FDOM) is the part of CDOM that emits fluorescence after absorbing ultraviolet and visible light [37]. In this study, an F-7000 fluorescence photometer (Hitachi Company) was used to measure the fluorescence excitation-emission matrices (EEMs) of the CDOM. The EEMs were then analyzed through Parallel Factor Analysis (PARAFAC) to determine the fluorescent components of FDOM, which, in turn, were used to determine the source of the CDOM [38]. The EEM measurements provide specific information on the CDOM, such as the maximum excitation/emission wavelength (Ex_max_/Em_max_) and fluorescence intensity. Moreover, fluorophores are useful in distinguishing CDOM material sources. EEMs are a useful tool for obtaining distinct “spectral fingerprints.” The three-dimensional fluorescence spectra were corrected by Raman scattering with ultrapure water. The PARAFAC was carried out using the drEEM toolbox (ver. 0.2.0) of MATLAB.

### 3.4. Measurements of Chl-a, SCC, and Other Water Quality Parameters

Additional water quality parameters were measured, including Chl-a, SSC, and salinity. Chl-a was measured by spectrophotometry (GB 17378.7-2007). For each sample, the phytoplankton pigment was extracted with acetone solution, and the absorbance was measured at a wavelength of 664 nm to determine the Chl-a content.

SSC was measured by the gravimetric method (GB 17378.4-2007). Each water sample was filtered through a 0.45 µm filter membrane, and the SSC was quantified by subtracting the weight of a dried blank filter membrane from the weight of the dried filter membrane containing the filtered suspended matter.

The salinity was measured using an LYT-610 salinity meter (GB 17378.4-2007). For these measurements, first, the conductivity ratio of the water sample to standard seawater was measured, and then common tables on international oceans were queried to obtain the salinity of the water samples. The measured data are shown in Table 2.

#### Water Surface Spectra

Water surface spectra were collected to obtain optical parameters [39]. An AvaField-2 portable hyperspectral spectrometer (Avantes Company, The Netherlands) was used to collect the water surface spectra over a wavelength range of 300–1700 nm and with a spectral resolution of about 1.4 nm. At least three measurements were taken at each station, and at least 10 spectral curves were detected each time. The radiation values of the sky light and whiteboard were also measured. The reflectance was calculated using the following formula:

(2) $$Rrs=\frac{\left(Lu-Ls*\rho f\right)*Rp}{Lp*\pi },$$

where *Rrs* is the remote sensing reflectance (Sr^−1^); *L_u_* is the radiance of the water surface (W·m^−2^·nm^−1^·Sr^−1^); *L_s_* is the radiance of the sky light (W·m^−2^·nm^−1^·Sr^−1^); *ρf* is the Fresnel coefficient of the water surface, taking the empirical value of 0.02; *R_p_* is the reflectance of the standard plate; and *L_p_* is the radiance of the standard plate (W·m^−2^·nm^−1^·Sr^−1^). It was necessary to resample each spectrum until a resolution of 1 nm was obtained.

For the roughness of the sea surface, it was necessary to denoise the spectral curve obtained from the in situ measurement [40,41]. In this study, the wavelet transform method was used to denoise spectral curves. Using MATLAB software, the ddencmp function was applied to generate the default threshold of the signal, and the wdencmp function was used [33].

## 4. Data and Results

### 4.1. Remote Sensing Images Processing

#### 4.1.1. Chl-a Inversion Algorithm

The band (622.486nm) and band ratio (622.486/851.341) are selected for their highest correlation with the Chl-a, and linear, exponential, logarithmic, and quadratic algorithms are constructed to obtain Chl-a concentration. The comparison shows that the linear band ratio model has the highest degree of fit, and the formula is as follows:

(3) $${\mathrm{Chla}}_{\;\left(CASI\right)\;}=0.9601\times \left(\frac{Rrs(622.486)}{Rrs(851.341)}\right)+0.0739$$

where *Rrs* (622.486) and *Rrs* (851.341) represent the remote sensing reflectance of CASI data center at 622.486 nm and 851.341 nm, respectively. The average relative error between the inversion value and the measured value is 14.77%, and there was a significant correlation between the inversed and measured Chl-a data (*p* < 0.01, R^2^ = 0.81).

In order to maintain consistency with the aerial remote sensing inversion model, the MODIS data of this study also adopted the band ratio model for the inversion of Chl-a concentration:

(4) $${\mathrm{Chla}}_{\;\left(MODIS\right)\;}=0.9117\times \left(\frac{Rrs(645)}{Rrs(858)}\right)+0.0648$$

where *Rrs* (645) and *Rrs* (858) represent the reflectance of the red band at 645 nm and the near-infrared band at 858 nm, respectively. The average relative error between the inversion value and the measured value is 16.73%, and it shows significant correlation between the inversed and measured data (*p* < 0.01, R^2^ = 0.78).

#### 4.1.2. SSC Inversion Algorithm

As indicated by the correlation analysis, the correlation coefficient between the reflectance and SSC at 622.62 nm is the highest. With this band as the dependent variable, the linear, exponential, logarithmic, and quadratic relational models of the single band and band ratio were constructed to retrieve the concentration of SSC. The results show that the single band index model has the highest degree of fit, and the specific formula is as follows:

(5) $${\mathrm{SSC}}_{\left(CASI\right)}=4.829\times \exp\left(19.619\times Rrs\left(622.486\right)\right)$$

where *Rrs* (622.486) represents the remote sensing reflectance at the CASI wavelength of 622.486 nm. The average relative error between the inversion value and the measured value is 11.16%, and a significant correlation between the inversed and measured SSC (*p* < 0.01, R^2^ = 0.96) is observed.

In order to maintain consistency with the CASI inversion algorithm, a single-band algorithm was also adopted for the SSC inversion using MODIS data. The formula is as follows:

(6) $${\mathrm{SSC}}_{\left(MODIS\right)}=4.829\times \exp\left(19.619\times Rrs(645)\right)$$

where *Rrs* (645) represents the reflectance of the MODIS in the red band of 645 nm. The average relative error between the inversion value and the measured value is 10.18%, and there was a significant correlation between the inversed and measured SSC (*p* < 0.01, R^2^ = 0.92).

### 4.2. Chl-a Concentration

**Figure 4:**
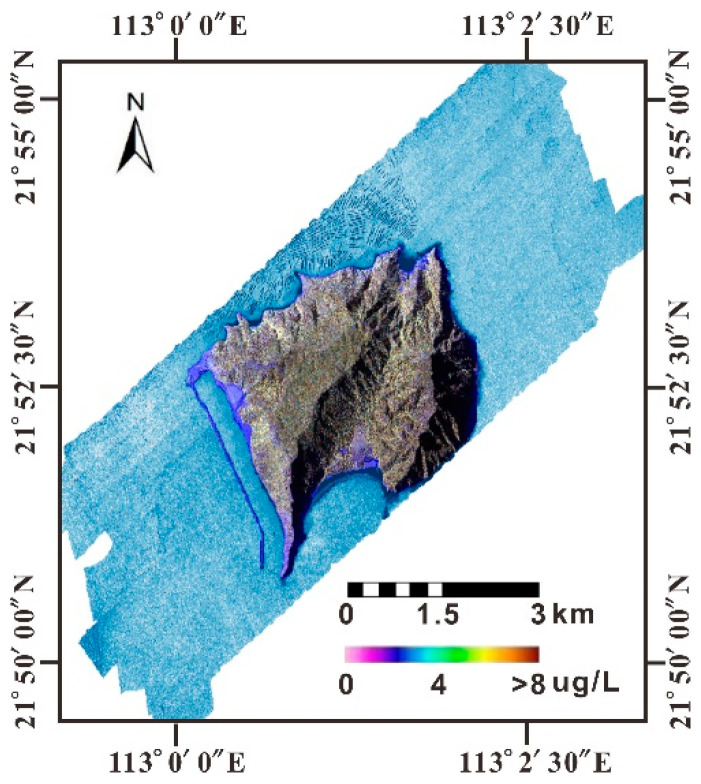
Distribution of Chl-a inversed by CASI data in the sea area around Dajin Island.

**Figure 5:**
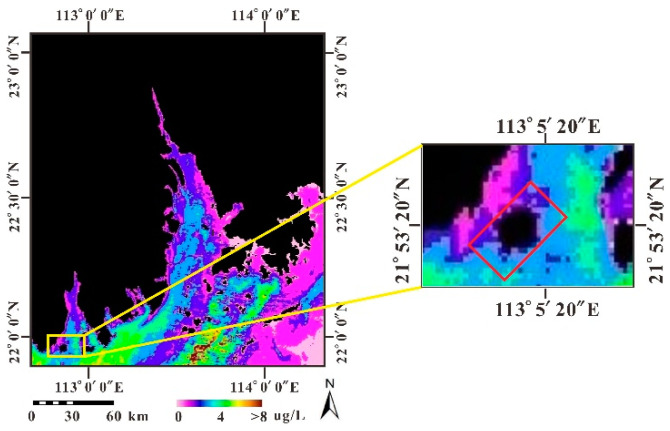
Distribution of Chl-a inversed by MODIS data in the Pearl River Estuary.

Chl-a is evenly distributed in the study area with low concentrations ranging from 0.1–4.4 μg/L (Figure 4), which is consistent with the observation by the MODIS, as shown in Figure 5.

However, Chl-a concentrations in the Pearl River Estuary fluctuate over a wider range from lower than detection to approximately 8.2 μg/L. The Chl-a gradually increases with the distance offshore. The high Chl-a shows clockwise stripped distribution outside the estuary, which may be due to coastal currents flow in a clockwise direction under the Coriolis force. It is generally believed that the concentration of Chl-a reflects the biomass of phytoplankton. Usually, in the sea area where the saline and fresh water are fully mixed, phytoplankton boost under the suitable temperature, salinity, and nutrient concentration, which results in the increase of Chl-a concentration. However, the turbulent hydrodynamical environment and low transparency for the turbidity or predation by zooplankton may reduce the Chl-a in waters.

### 4.3. SSC Distribution

**Figure 6:**
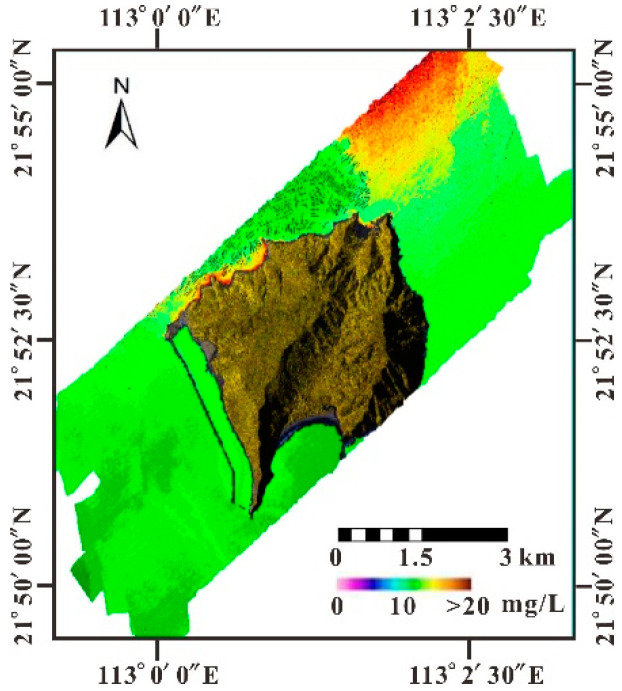
Distribution of SSC inversed by CASI data in the sea area around Dajin Island.

The SSC in the sea area around Dajin Island fluctuates within the range of 0.48–12.15 mg/L, as shown in Figure 6 based on CASI data. Higher SSC are distributed north of the island and along the northeast and northwest coasts, while the aquaculture area in the north has comparably lower SSC.

**Figure 7:**
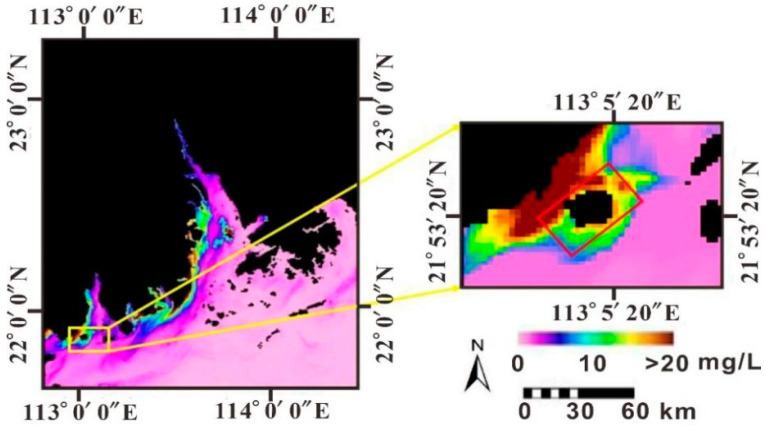
Distribution of SSC inversed by MODIS in the Pearl River Estuary (the red box is the coverage of the CASI image).

According to MODIS/SSC (Figure 7), the SSC in the Pearl River Estuary varies from 0.03–20.45 mg/L, which is consistent with previous studies [42,43]. A high SSC distributes along the coastline and exhibits zonal decrease with distance offshore from the northeast to the southwest. Moreover, MODIS/SSC indicated that the unique geographical location and hydrodynamics are the main factors impacting the SSC distribution around Dajin Island. Once the land-sourced SSC enters the sea area of the northern Dajin Island, the SSC along the west bank of the Huangmao Sea becomes significantly higher than that along the east bank due to the deflection force of the geostrophic force. The obstruction caused by Dajin Island leads to an obvious branching phenomenon north of the island, which results in a higher SSC along two sides of the island. The static hydrodynamic environment that developed north of the island and that is characterized by a low SSC (and high transparency) is suitable for aquaculture. Generally, SSCs gradually decrease to the southeast and southwest of Dajin Island.

### 4.4. DOC Distribution Coupled with CDOM Optical Properties

The distribution of DOC and CDOM optical properties are analyzed along with a comparison with salinity measurements (see Table 2) to obtain more substantial information on the supply of organic materials for local primary production.

**Figure 8:**
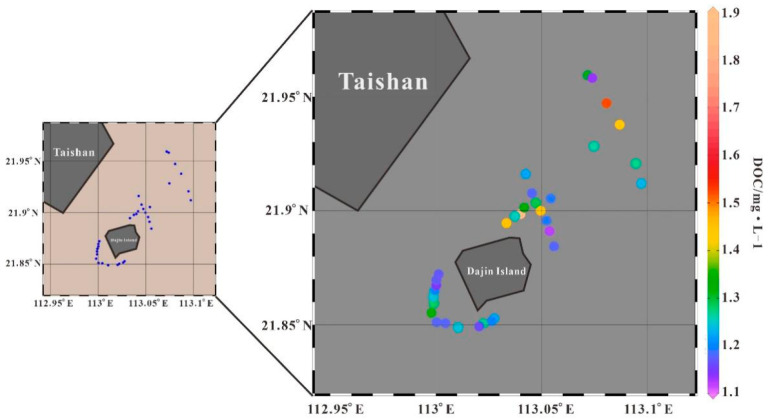
Distribution of measured DOC concentrations.

**Figure 9:**
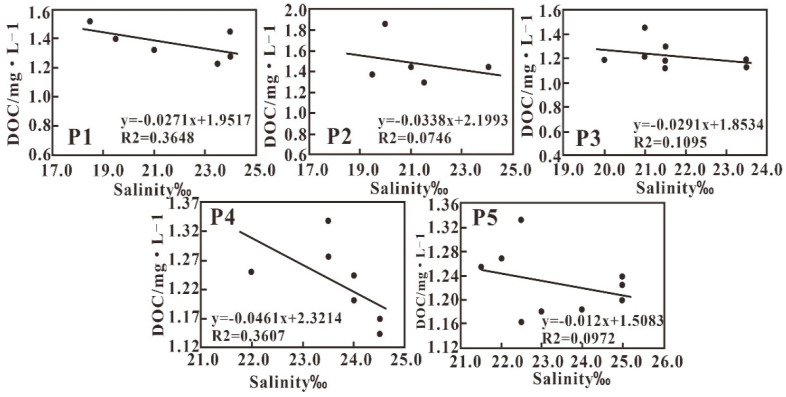
The correlation between DOC and salinity along the five transects.

The DOC in the study area varies between 1.12 mg·L^−1^ and 1.85 mg·L^−1^, with an average value of 1.29 mg·L^−1^. High DOC concentrations are distributed northeast of the island and are possibly related to terrestrial sources and aquaculture inputs (Figure 8). DOC and salinity show a negative correlation in each transect (Figure 9), indicating that the input of dissolved organic matter in this area is mainly controlled by land-sourced runoff, and its concentration change is controlled by a dilution and diffusion mechanism. The weak correlation between DOC and salinity along transect P5 may be due to the release of DOC during resuspension under the tide. P2 is the section connecting P1 with aquaculture showing high DOC with a narrow salinity variation and weak correlation.

#### 4.4.1. The Absorption Coefficient, α (441), and Its Correlation with Salinity

**Figure 10:**
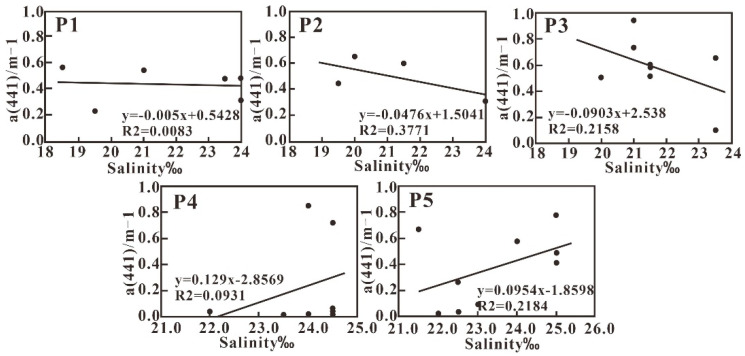
Variations in α (441) with salinity along the five transects.

A distinct correlation between salinity and the CDOM absorption coefficient has been suggested especially in coastal waters where land-sourced organic matter is considered as the main source of CDOM [44,45]. The α (441) in the study sea area ranges between 0.0125 m^−1^ and 0.8544 m^−1^, with an average value of 0.4874 m^−1^. This value is consistent with that reported for the eastern channel of Xiamen Bay [46], but is much lower than the value reported for the Pearl River Estuary [47]. In general, Transect P1, P2, and P3 are characterized by relatively high α (441) and low salinity; however, the weak negative correlation between α (441) and salinity is indicative of the complex diffusion procedure of fresh runoff with saline marine water. In addition, α (441) along transect P4 and P5 exhibits great fluctuation (Figure 10). The resuspension of sediment could be the main factor impacting α (441) and its relationship with salinity. In addition, the input of various organic compositions may be another reason inducing the deflection of conservative behavior.

#### 4.4.2. Fluorescence Intensity, Fs (441), and Its Correlation with Salinity

**Figure 11:**
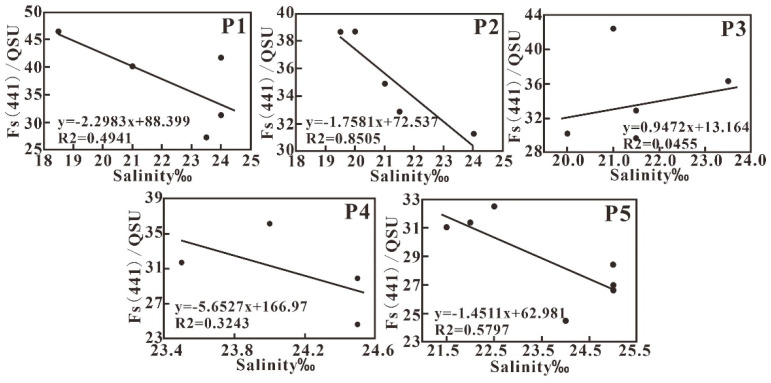
Variations in Fs (441) with salinity along the five transects.

A negative correlation between fluorescence intensity Fs (441) and salinity in the studied sea area is observed (Figure 11), which indicates the mixing behavior of CDOM in water. Further analysis showed that the correlation between Fs (441) and salinity varies between different transects except P3 transect, which shows a weak positive correlation. The extra input of CDOM through resuspension of sediment should be the main factor impacting the relationship between Fs (441) and the salinity of P3 transect. Usually, the fluorescence intensity is more efficient in indicating CDOM with various compositions.

## 5. Discussion

### 5.1. Eutrophication Evaluation

Chl-a is an essential parameter used to indicate a water body’s eutrophication status [48]. The Carlson eutrophic index (TSI) based on Chl-a is calculated as follows:

(7) $$\mathrm{TSI}=10\times \left(6-\frac{2.04-0.68\ln(\text{Chl-a})}{\ln2}\right)$$

TSI is divided into four grades within the range of 0–100: oligotrophic (TSI < 30, Chl-a < 2.6), mesotrophic (40 ≤ TSI < 50, 2.6 ≤ Chl-a < 8.9), eutrophic (50 ≤ TSI < 70, 8.9 ≤ Chl-a < 52), and extremely eutrophic (70 ≤ TSI, 52 ≤ Chl-a).

**Figure 12:**
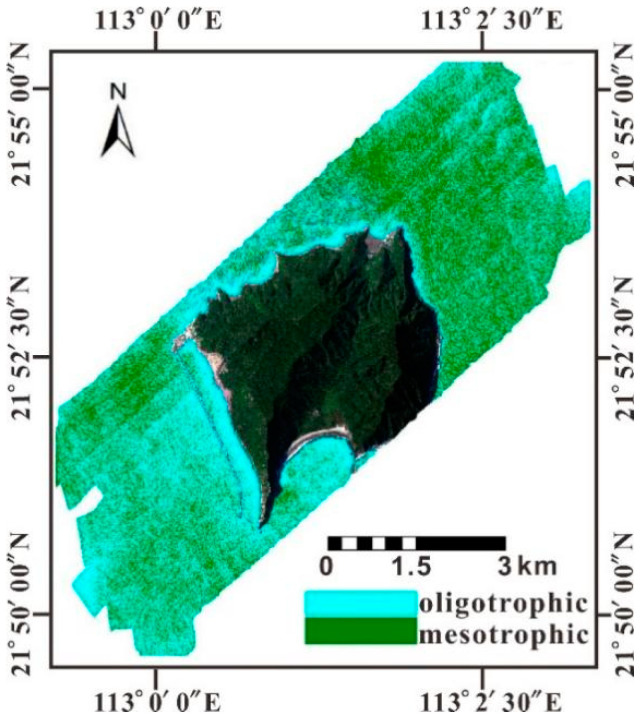
Image depicting the eutrophication status of the sea area around Dajin Island.

According to the assessment, the sea area around Dajin Island shows an oligotrophic-mesotrophic eutrophication level in general. Particularly, the aquaculture area in the north of the island is oligotrophic (Figure 12). The eutrophication status evaluated with TSI is contradictory to the local environmental bureau’s water quality assessment, which indicated that the Huangmaohai Sea is seriously polluted by inorganic nitrogen and reactive phosphorus. The potential inhibition of phytoplankton growth by turbidity is analyzed with inverted SSC by the CASI and MODIS.

Although no obvious correlation between SSC and Chl-a is observed in the study area, SSC in the Pearl River Estuary indicated an inverse correlation with Chl-a in the Huangmaohai Sea. Thus, the low Chl-a in the sea area around Dajin Island may be induced by the high SSC with low transparency. In addition, the Huangmao Sea is an estuary with weak runoff but very strong reciprocating tidal flow. The measured maximum average flow velocity of the high tide flowing to the northeast is 1.24 m/s, and the maximum flow velocity is supposed to be 1.01 m/s at the surface [49]. The turbulent hydrodynamical environment may result in low biomass, even though nutrient availability is high. Considering the high Chl-a concentration in the outer estuary inversion by MODIS/Chl-a image, it should be anticipated that the abundant nutrients carried by runoff from land source and released from aquaculture activities will incur algal blooms under suitable conditions. Therefore, eutrophication may be underestimated based on the Chl-a. The study area is deduced to be in mesotrophic state.

It should be noted that accurate acquisition of Chl-a and SSC is important in the correct evaluation of the water environment and the proposal of targeted environ-mental protection measures [50,51]. Due to the huge differences of aquatic environments in the coastal water of China, the establishment of a regional ocean color inversion model is necessary.

### 5.2. Material Sources Tracing

#### 5.2.1. The Spectral Slope Coefficient, Sg, for the Assessment of Water Quality Parameters

Sg is closely related to the composition of CDOM [52]. The ratio of humic acids to fulvic acids greatly affects the value of Sg. The higher the ratio, the larger the molecular weight of CDOM and the smaller the Sg [53,54]. Sg was fitted with the regression exponential model of CDOM absorption:

(8) α(λ)=α(441)exp(Sg(441−λ))−0.0645

Due to the significant proportion of humic acids imported from terrestrial sources, the coastal water is characterized by relatively high absorption but lower Sg [53], resulting in a negative correlation between the absorption coefficient and Sg.

Sg in the study area varies between 0.0067 nm^−1^ and 0.0335 nm^−1^, with an average value of 0.0123 nm^−1^, and is basically the same as that measured in the waterway at the east of Xiamen Bay [46]; however, it is much lower than that measured in the Pearl River Estuary [47].

**Figure 13:**
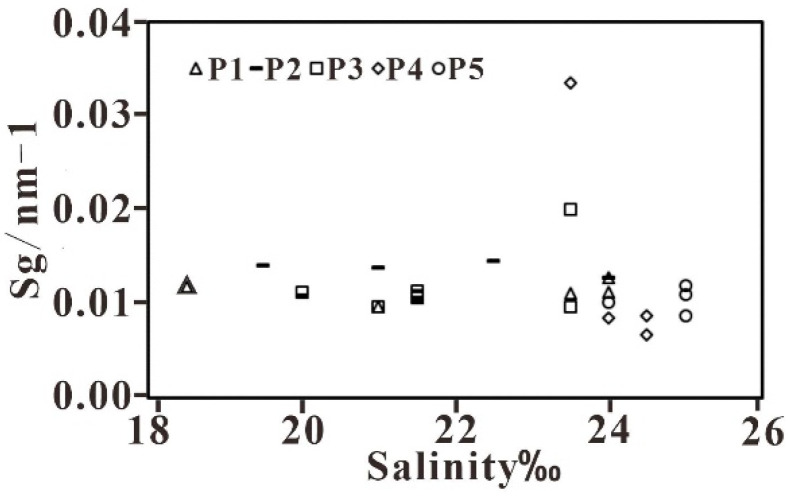
The relationship between Sg and salinity along the five transects.

There is no obvious correlation between Sg and salinity. It should be noted that Sg varies within a narrow range, while salinity ranges between 18.5 and 25‰ (Figure 13), which indicates that the composition of CDOM in this sea area is monotonous. Given that Sg in the study area is relatively low compared to other estuaries (for example, the Sg in the Yangtze Estuary is 0.0115–0.0175 nm^−1^ [47]), it is inferred that the CDOM composition in this sea area is controlled by terrestrial runoff [55].

#### 5.2.2. CDOM Fluorescence Properties

CDOM fluorescence spectroscopy is often used to analyze dissolved organic substances in water and trace the material sources. Besides the degradation products of terrestrial plants, organic soils, and the discharge of wastewater, CDOM can be produced in natural water due to biological excrement, biological degradation of aquatic plant remains, and other processes [40,41]. Excitation emission matrix spectroscopy (EEM) can be used to perform spectral identification and characterize the overlapping fluorescence spectra for multi-component systems, so as to extract information on CDOM components.

**Figure 14:**
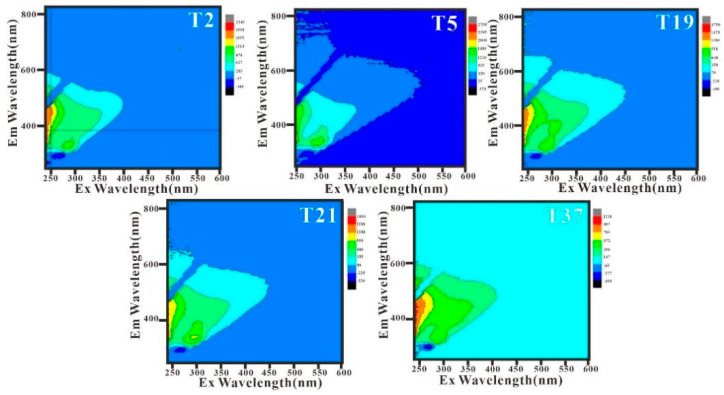
Three-dimensional fluorescence spectra of representative stations.

Considering the similarity in fluorescence spectroscopy between the different stations of each transect, one representative station was selected for discussion from each transect, as shown in Figure 14.

The fluorescence spectra obtained in this study were divided into the following regions: region I (Ex/Em = 240–250 nm/250–330 nm), region II (Ex/Em = 240–250 nm/330–380 nm), region III (Ex/Em = 240–250 nm/380–825 nm), region IV (Ex/Em = 250–600 nm/240–380 nm), and region V (Ex/Em = 250–600 nm/380–825 nm). These represent aromatic protein tyrosines, aromatic white acid substances, fulvic acids, soluble microbial metabolites, and humic acids, respectively [35].

Humic and fulvic acids are humic acid-like components, and tryptophan and tyrosine are protein-like components. Usually, humic acid-like components mainly come from soil and decaying plant debris from land sources, while protein-like components are mainly produced by biological processes, including degradation by bacteria and microorganisms, or from industrial and agricultural wastewater and domestic sewage. Thus, the fluorescence intensity of tryptophan can also be used as an indicator of water bodies that have been heavily affected by human activities [56,57].

There are two obvious fluorescence peaks, A and B, in the fluorescence spectrum of the study sea area, which appear in region II and region IV, respectively. The maximum fluorescence intensity is used to characterize the corresponding fluorescence value (standardized fluorescence unit QSU), which corresponds to amino acid protein substances and soluble microbial metabolites. Among these, the characteristic area of the aromatic white amino acid substance, which is represented by fluorescence peak A, is obvious; contrarily, the characteristic area of soluble microbial metabolites represented by fluorescence peak B is not obvious and presents with a wider but lower peak. Therefore, it can be inferred that the organic materials in the study area mainly originate from terrestrial sources and aquaculture areas.

### 5.3. Relationship between DOC and CDOM Optical Properties

Traditionally, DOC is measured based on fixed-point sampling and laboratory analysis with high accuracy; however, it is time-demanding and costly. Several studies have shown the capability of the CDOM absorption coefficient in quantifying the DOC concentration in offshore waters; particularly, regional relationships can be established to invert the terrestrial DOC input [47]. However, the relationship has great uncertainty depending on the region, the season, and hydrodynamics [58,59].

Compared with other estuaries in the world, the concentrations of DOC and CDOM measured in the study area are relatively low. This may be due to the low vegetation coverage in the Pearl River Basin [44] and the wide application of inorganic fertilizers in farmland [59]; the high turbidity which inhibits the phytoplankton fluorosis is another important reason.

#### 5.3.1. Correlation Analysis of DOC and α (441)

**Figure 15:**
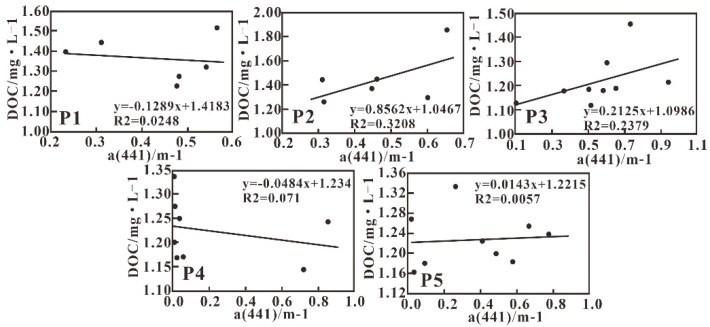
Relationship between DOC and α (441).

A weak positive correlation between DOC and CDOM is visible in the studied sea area (Figure 15). Further analysis showed that the correlation between DOC and α (441) varies between different transects; in particular, the P2, P3, and P5 showed a weak positive correlation, while the P1 and P4 transects showed a weak negative correlation. The weak and different correlation on different sections indicated that it is difficult to quantify DOC using CDOM absorbance properties.

Usually, the weak correlation between DOC and α (441) can be attributed to the diversity of DOC sources resulting in various DOC compositions, which leads to complicated CDOM optical properties [60]. DOC from terrestrial sources contains more colored parts, while colorless DOC accounts for a greater proportion of DOC from the degradation of phytoplankton [61]. Furthermore, DOC composed of soil humic and fulvic components is relatively stable and not easily decomposed. However, the photochemical effect can convert marine autogenous CDOM into noncolored DOM [54]. In addition, the Xijiang River collects water from different tributaries, each having its own unique optical characteristics, which leads to complex correlation between CDOM and DOC and the unavailability of a good linear relationship [59]. Moreover, as both CDOM and DOC vary in narrow ranges in a small sea area, it is difficult to get good correlation. Therefore, an expanded study area is suggested for future investigations to obtain a feasible regional algorithm of DOC by CDOM absorbance coefficients.

#### 5.3.2. Correlation Analysis of DOC and Fs (441)

**Figure 16:**
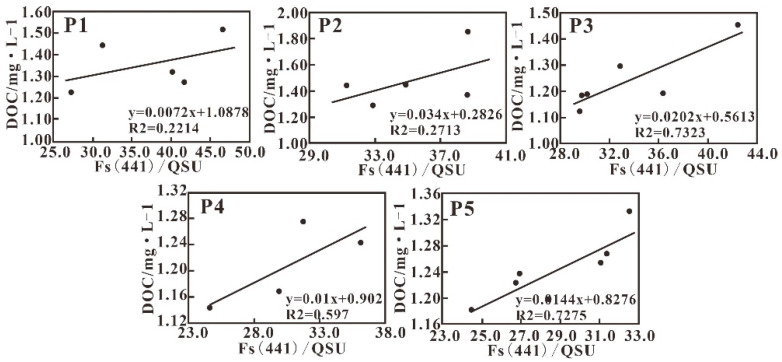
The relationship between fluorescence intensity and DOC along each transect.

The fluorescence intensity Fs (441) shows a stronger positive correlation with DOC concentration along each transect (Figure 16). It has been concluded that in this hydrodynamically complicated area, fluorescence is more informative than absorption. Using the fluorescence intensity of different fluorophores to quantify the amount of CDOM from different sources with various compositions appears promising; however, this method requires further study.

## 6. Conclusions

Based on the remote sensing images obtained with CASI and MODIS, this paper studied the distribution of Chl-a and SSC around Dajin Island in the Pearl River Estuary and evaluated the state of eutrophication around Dajin Island using Chl-a inversion results. Meanwhile, using DOC/CDOM data from the study area, DOC composition and material source tracing were performed to achieve a comprehensive assessment of the environmental quality and ecological risk of the Dajin Island marine area.

The distribution characteristics of the DOC and CDOM absorptive and fluorescent properties all indicate that terrestrial input is the main material source of DOM in the study area; however, the DOM produced by aquaculture cannot be ignored. Dilution with saline marine water is an important factor impacting the regional variation in DOC and a (441), especially for transects P1, P2, and P3.

Although the eutrophication assessment based on CASI/Chl-a indicated that the study sea area is in an oligotrophic-mesotrophic state, the water body around Dajin Island is notably rich in DOM. The higher Chl-a in the outer estuary inversion from MODIS further indicated that the number of nutrients supports the phytoplankton to flourish in suitable environment. The sea area around Dajin Island is, thus, deduced to be in a mesotrophic state despite a low Chl-a concentration. The high turbidity and complex hydrodynamic are indicated to be the cause of low Chl-a, which leads to the underestimation of eutrophication.

Both DOC and CDOM vary in narrow ranges with a low variation gradient of salinity in limited space coverage, which induce the failure of relationships between DOC and CDOM optical properties. Therefore, an investigation that expands the scope of the research area is necessary to establish a regional inversion algorithm of DOC by CDOM optical properties. In addition, DOC related fluorescence properties are promising not only for quantifying DOC, but also for providing information on material sources, which is very important for implementing protection policies by the local government and, eventually, suitable water treatments.

This study used data from different platforms at different observational scales to perform ecological monitoring and determine impacting factors on eutrophication in the sea area around Dajin Island. Comprehensive long-term monitoring and ecological health assessments in the sea area surrounding Dajin Island need to be performed. Correlations between DOC and CDOM optical properties need more investigation in this area to assess the possibility of establishing a commonly accepted DOC inversion algorithm by CDOM through remote sensing.

## Figures and Tables

**Table 1:** The technical parameters of CASI 1500 h in comparison with MODIS.

	Sensor System	CASI 1500 h	MODIS
Parameter			
Spectral range/nm		380–1050	400–1440
Number of spectral channels		Up to 288, customizable settings	36
Spectral resolution/nm		<3.5	/
Spatial resolution/m		0.6	250, 500, 1000
Scanning width/km		About 0.3	2330
Data rate/MB/sec		19.2	/
Pixel size/µm		20 × 20	/
Spot size pixels		<1.5	/

**Table 2:** Measured results of the main parameters in the study area.

Station	SSC (mg/L)	Chl-a (ug/L)	DOC (mg/L)	Salinity (‰)	Station	SSC (mg/L)	Chl-a (ug/L)	DOC (mg/L)	Salinity (‰)
T1	10.07	0.21	1.40	19.5	T20	11.34	1.74	1.22	25
T2	7.54	0.82	1.37	19.5	T21	3.54	1.69	1.20	25
T3	8.74	0.96	1.85	20	T22	13.14	1.36	1.27	22
T4	8.34	2.09	1.45	21	T23	3.54	2.78	1.16	22.5
T5	9.54	0.83	1.32	21	T24	2.74	1.61	1.33	22.5
T6	6.14	1.56	1.52	18.5	T25	7.34	0.73	1.25	21.5
T7	1.77	1.93	1.44	24	T26	6.54	3.35	1.24	25
T8	6.94	2.14	1.26	--	T27	3.74	3.35	1.18	23
T9	1.87	2.41	1.27	24	T28	10.34	3.35	1.18	24
T10	7.87	3.35	1.23	23.5	T29	8.74	3.35	--	--
T11	10.94	1.01	1.22	21	T30	2.74	3.35	1.34	23.5
T12	10.94	1.12	1.18	21.5	T31	2.34	1.91	1.28	23.5
T13	11.14	0.89	1.29	21.5	T32	10.54	1.32	1.24	24
T14	3.26	0.82	1.45	21	T33	8.14	1.11	1.25	22
T15	11.74	0.21	1.19	20	T34	9.74	0.55	1.2	24
T16	10.34	0.37	1.12	21.5	T35	8.74	2.69	1.14	24.5
T17	10.74	0.70	1.18	--	T36	7.54	1.98	1.17	24.5
T18	4.34	1.57	1.13	23.5	T37	9.94	1.88	1.17	24.5
T19	2.14	2.46	1.19	23.5	/	/	/	/	/

Note: -- refers to the missing data.

## Data Availability

The data contained in the paper are available from the authors.
